# High spectro-temporal compression on a nonlinear CMOS-chip

**DOI:** 10.1038/s41377-021-00572-z

**Published:** 2021-06-18

**Authors:** Ju Won Choi, Ezgi Sahin, Byoung-Uk Sohn, George F. R. Chen, Doris K. T. Ng, Anuradha M. Agarwal, Lionel C. Kimerling, Dawn T. H. Tan

**Affiliations:** 1 Photonics Devices and System Group, SUTD-MIT International Design Center, Singapore University of Technology and Design, Singapore, 487372 Singapore; 2 Institute of Microelectronics, A*STAR (Agency for Science, Technology and Research), 2 Fusionopolis Way, #08-02, Innovis Tower, Singapore, 138634 Singapore; 3 Microphotonics Center, Massachusetts Institute of Technology, 77 Massachusetts Avenue, Cambridge, MA 02139 USA; 4 Materials Research Laboratory, Massachusetts Institute of Technology, 77 Massachusetts Avenue, Cambridge, MA 02139 USA; 5 Department of Materials Science and Engineering, Massachusetts Institute of Technology, 77 Massachusetts Avenue, Cambridge, MA 02139 USA

**Keywords:** Optical materials and structures, Nonlinear optics

## Abstract

Optical pulses are fundamentally defined by their temporal and spectral properties. The ability to control pulse properties allows practitioners to efficiently leverage them for advanced metrology, high speed optical communications and attosecond science. Here, we report 11× temporal compression of 5.8 ps pulses to 0.55 ps using a low power of 13.3 W. The result is accompanied by a significant increase in the pulse peak power by 9.4×. These results represent the strongest temporal compression demonstrated to date on a complementary metal–oxide–semiconductor (CMOS) chip. In addition, we report the first demonstration of on-chip spectral compression, 3.0× spectral compression of 480 fs pulses, importantly while preserving the pulse energy. The strong compression achieved at low powers harnesses advanced on-chip device design, and the strong nonlinear properties of backend-CMOS compatible ultra-silicon-rich nitride, which possesses absence of two-photon absorption and 500× larger nonlinear parameter than in stoichiometric silicon nitride waveguides. The demonstrated work introduces an important new paradigm for spectro-temporal compression of optical pulses toward turn-key, on-chip integrated systems for all-optical pulse control.

## Introduction

The ability to control an optical pulse’s temporal and spectral properties is an important function. Temporal compression of pulses impacts the capacity of optical information systems^1,2^, the resolution of metrology tools^3^ and bioimaging techniques^4,5^, while spectral compression provides design degrees of freedom for high brightness spectroscopy^6^ and augmented control in all-optical signal processing^7^. In many of these applications, system compactness, compatibility with CMOS-electronics and good power/energy efficiency are often advantageous for practical deployment; the merits of completely chip-scale systems for the manipulation of pulse properties are elucidated by a number of demonstrations of on-chip temporal compression of optical pulses^8–17^. One of the first reports of optical pulse compression on a CMOS chip was reported by Moss et al.^8^, where 1.6× compression of optical pulses was demonstrated in a 45 cm long Hydex waveguide. This was closely followed by a report of 7× compression on a silicon chip, albeit with low power efficiency, exhibited by a low, 1.8× increase in the output peak power of the pulse^9^. Since then, various CMOS-chip based optical pulse compression schemes have been demonstrated, largely based on soliton-effects including: adiabatic soliton compression^8,11^, high-order soliton compression^10,12,15,16^, and Bragg soliton compression^17^. In these soliton-based compression schemes, the nonlinearity required for creation of new frequencies and the anomalous dispersion needed for temporal synchronization arise simultaneously along the entire device. Temporal compression may also be achieved using systems that separate the nonlinear and dispersive stages. For the latter, dispersive effects are concentrated in a strongly dispersive device, similar to those used for chirped pulse amplification systems^18,19^, or dispersion compensation in lightwave communication systems^20,21^. Such systems have the potential to achieve pulses with lower pedestals^22^ as dispersion can be independently manipulated for temporal synchronization. Importantly, the ability to independently design the nonlinear and dispersive properties provides greater flexibility in compressing pulses of various initial temporal widths.

To date, much of the progress on chip-based compression has focused on the temporal domain. To bring forth additional functionalities to pulse control, a single system that allows both temporal and spectral pulse compression would provide a key advancement toward an integrated, flexible solution for independently controlling the pulse’s temporal and spectral properties. Pulse spectrum reduction is typically achieved by bandpass filtering of selected spectral components of the pulses, a process that results in loss of pulse energy. Alternatively, to conserve the pulse energy, self-phase modulation (SPM) of pre-chirped pulses may be manipulated to reduce the pulse spectrum. Such spectral compression has been implemented in fiber^23–25^ but has been more elusive in chip-scale platforms. Importantly, a system where both spectral and temporal compression can be activated independently on demand, allows pulses to be temporally expanded or compressed while possessing a spectrum that approaches the pulse transform limit. Such a system can facilitate tunability in lasers, providing a means to flexibly derive temporally short or long pulses as needed by the application.

In this paper, we demonstrate an all-optical integrated pulse compression system that is capable of generating strong temporal compression of pulses as well as energy-efficient spectral compression. The system is implemented on the two-photon absorption (TPA)-free, CMOS-compatible ultra-silicon-rich nitride (USRN) platform, which possesses 500× larger nonlinear parameter than that in stoichiometric silicon nitride waveguides. Importantly, we demonstrate high temporal compression of 5.8 ps pulses to 0.55 ps, equivalent to a compression factor of 11× using a low input peak power of 13.3 W, as well as 9.4× increase in the pulse peak power. This represents one of the highest performing on-chip optical pulse compressors to date. In addition, we report the first demonstration of on-chip spectral compression of optical pulses: 480 fs pulses at a peak power of 14 W are spectrally compressed by 3.0×. The USRN platform (i) enables high field localization in the guided wave medium that allows for low power compression, (ii) shows negligible TPA and large Kerr nonlinearity at 1550 nm, features that are ideal for efficient compression, and (iii) preserves the pulse energy during the spectral compression process, such that sufficient nonlinear frequency shifts can occur even after dispersive broadening acts on the pulse. Another key enabler of the spectro-temporal compressor is the dispersive stage which possesses highly linear group delay, large bandwidth and high transmissivity, as well as the ability to accurately engineer the magnitude of anomalous dispersion in the USRN compressor. The results demonstrate an integrated, CMOS-compatible system with the unique capability of effecting good spectral compression or strong temporal compression interchangeably by appropriate choice of the input and output ports.

### Principles of dual-stage spectro-temporal compression

**Fig. 1:**
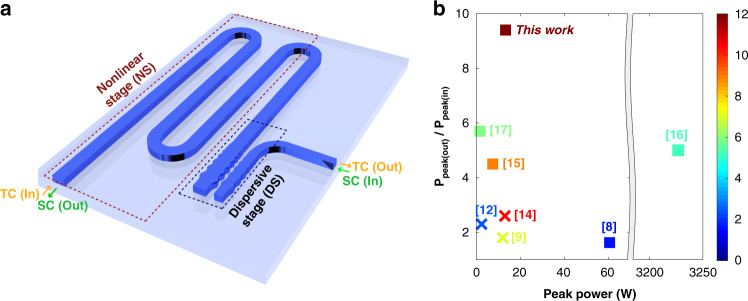
Schematic of the USRN compressor system and comparison with other CMOS-chip based temporal compressors. **a** Schematic of the USRN compressor system. The system is comprised of separate nonlinear and dispersive stages. For spectral compression (SC), pulses enter the dispersive stage (DS) first before the nonlinear stage (NS), and for temporal compression (TC), pulses enter NS first before DS. **b** Comparison plot for various CMOS-chip based temporal compressors, comparing *P*_peak(out)_/*P*_peak(in)_, compression factors and peak powers used for compression. Squares (■) denote TPA-free compression, crosses (×) denote that TPA is present, and the color bar denotes the temporal compression factor. For refs. ^8,12,16,17^, *P*_peak(out)_/*P*_peak(in)_ was not reported. For these, we have denoted the values of *P*_peak(out)_/*P*_peak(in)_ to be equivalent to the compression factor. In general, *P*_peak(out)_/*P*_peak(in)_ will be lower than compression factor because of the presence of pulse pedestals. In systems with TPA (**×**), this metric will be further reduced by nonlinear losses

**Fig. 2:**
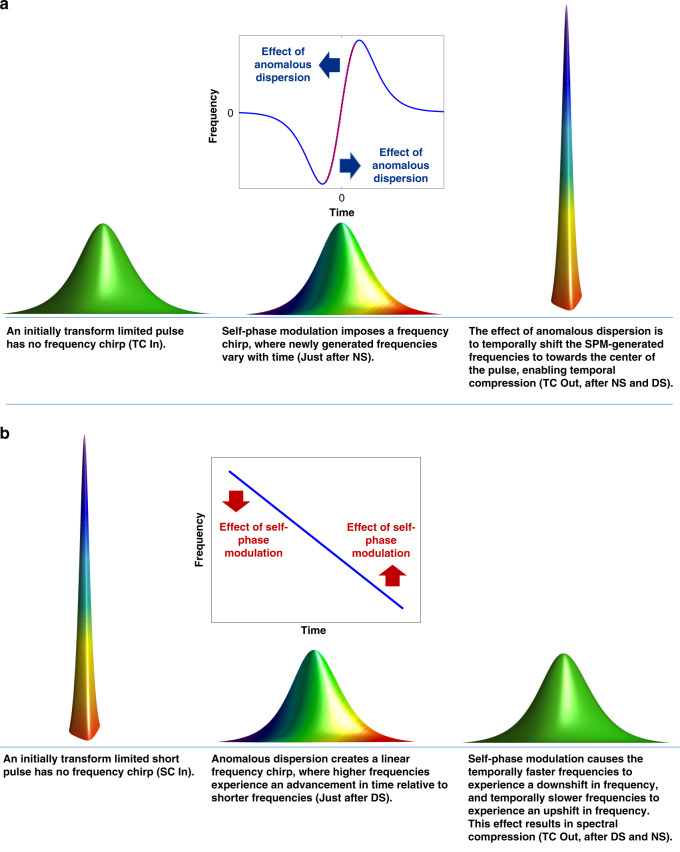
Principles of the nonlinear USRN pulse compressor. **a** Compression in the temporal domain involves the acquisition of new frequencies via SPM. In the second stage, anomalous dispersion temporally shifts the generated frequencies toward the center of the pulse, in so forth achieving temporal compression. **b** Compression in the spectral domain involves first applying anomalous dispersion to the pulse. This imparts a chirp to existing pulse frequencies. SPM comes in at the second stage and creates a frequency downshift in the temporally faster frequencies, and a frequency upshift in the temporally slower frequencies. Brackets in captions denote the location of the pulse with reference to Fig. 1

Fig. 1a shows the key elements comprising the pulse compression system: a nonlinear stage and a dispersive stage, and the operating principle is illustrated in Fig. 2. In the system for temporal compression, an initially transform limited pulse which is temporally long may be assumed to have minimal frequency chirp. On entering the first stage with high nonlinearity, the pulse if possessing sufficient peak power will undergo SPM (or in other cases, cross phase modulation may occur with a co-propagating pulse). The profile of the frequency chirp imposed by SPM is shown in Fig. 2a. Linear chirp is limited to the central part of the pulse and explains why solitons are often observed to possess pulse pedestals^22^. In the second stage, anomalous dispersion accelerates the slower moving blue frequencies and decelerates the faster moving red frequencies. This causes the newly generated frequencies to approach the center of the pulse, resulting in temporal compression.

Another key advantage of frequency chirp control through separate dispersive and nonlinear stages is the ability to harness their combined effects toward spectral compression^26–28^. An initially transform-limited short pulse possesses substantial frequency content and minimal frequency chirp. Pulses propagate through the dispersive stage first, and existing frequencies in the pulse experience a linear frequency chirp, the profile of which is shown in Fig. 2b. As a result, higher frequencies experience an advancement in time relative to the lower frequencies. Upon undergoing SPM, the temporally faster frequencies experience a frequency downshift whereas temporally slower frequencies experience a frequency upshift. As a result, spectral compression occurs. The first stage results in dispersive pulse broadening and a concomitant reduction in the pulse peak power. Consequently, the nonlinear efficiency of the platform is paramount to successful spectral compression.

In both temporal and spectral compression, the dispersive stage needs to have sufficiently large anomalous dispersion to match both the magnitude and generated profile of the chirp imparted by SPM. When considering on-chip implementations, achieving sufficiently high nonlinear phase shift at low pulse peak powers using waveguides is comparatively easier than achieving large magnitudes of dispersion. Waveguides on various platforms have been used to demonstrate efficient SPM at peak powers of several watts, including on silicon and chalcogenide platforms^29–32^. For example, a 10 ps pulse acquiring the same amount of spectral content through SPM as that of a transform limited 2 ps pulse would require temporal synchronization from dispersion on the order of several picoseconds.

To match the frequency chirp that is easily imparted through SPM, anomalous dispersive elements require significantly larger magnitudes than that achievable in photonic waveguides. A useful figure of merit for assessing dispersive elements is, $FOM_{{\rm{Dis}}} = \frac{{D.{\Delta} \lambda }}{\alpha }$ where *D* is the dispersion of the dispersion element, Δ*λ* is the operating bandwidth and *α* is the loss coefficient. Photonic waveguides have typical dispersion magnitudes of 1000 s of ps nm^−1^ km^−1^. If applying a 5 ps nm^−1^ delay on a pulse, waveguides of several hundred meters will be required, resulting in prohibitive losses. Contrary to photonic waveguides that have large bandwidth but small dispersion, structures like ring resonators and photonic crystal waveguides have comparatively large dispersion but insufficient bandwidths^33,34^. On-chip gratings are a promising approach to generate the magnitudes of anomalous dispersion required for both spectral and temporal compression. Theoretically governed by coupled-mode theory^35^, the imposition of a chirp in the grating pitch creates a variation in the path lengths traveled by the different frequencies of the pulse. Using both cladding-modulated and sidewall modulated structures, on-chip dispersion as large as −11.5 ps nm^−1^ and a group delay × bandwidth product of 218 ps have recently been demonstrated^36,37^. Our recent advancements in on-chip dispersion generation is a key enabler of this work, allowing us to accurately engineer the magnitudes of dispersion necessary for both high temporal compression and reversible spectro-temporal compression.

The fixed magnitude of anomalous dispersion in the dispersive element impacts the extent of compression achievable in the two-stage system: (1) for temporal compression, optimal compression occurs when the frequency chirp from SPM matches the dispersion in the grating. Therefore, provided sufficient nonlinear phase can be acquired, longer pulses have the potential for stronger compression. (2) Spectral compression on the other hand, relies first on dispersion induced temporal broadening. For a fixed dispersion, shorter pulses undergo a greater extent of temporal broadening, and have a higher potential for spectral compression, again provided sufficient nonlinear phase can be acquired in the second stage of the system. In the case of spectral compression, a tradeoff exists, where if the pulses are temporally broadened too much, the peak power of the pulse entering the USRN waveguide stage will be low, making it difficult to acquire sufficient nonlinear phase. In general, a smaller grating dispersion is useful for stronger temporal compression, whereas larger dispersion facilitates stronger spectral compression. This is provided sufficient nonlinear phase can be acquired in the nonlinear waveguide stage.

## Results

### Temporal and spectral compression dynamics

The design of the integrated pulse compression system is shown in Fig. 1a. The nonlinear stage is composed of a USRN waveguide with a length, width, and height of 5.5 mm, 450 nm, and 330 nm, respectively and SiO_2_ over- and under-cladding. USRN waveguides have been demonstrated to have good nonlinear optical performance, possessing a large Kerr nonlinearity and negligible nonlinear losses at 1550 nm^17,38–41^, (Supplementary Information 5). The dispersive stage is implemented using two sinusoidally varying waveguides with raised cosine apodization, placed side by side with a gap of 70 nm; the width of the waveguides (without the sinusoidal modulation) is 550 nm and 450 nm, with peak modulation amplitude of 50 nm and 35 nm, respectively.

The dynamics of pulse propagation in the integrated compression system are governed by the nonlinear Schrödinger equation (NLSE)^42^. Simulations of the NLSE are first performed to study the pulse compression system for both spectral and temporal compression. We study three different devices: (1) *Device 1*, defined as systems possessing a dispersive stage with *β*_2(grating)_ = −600 ps^2^ m^−1^. This value of dispersion is optimized for the purpose of demonstrating high temporal compression. In this design, the period, *Λ* of the dispersive stage experiences a linear increase, Δ*Λ* = 8 nm over the grating length of 500 μm. (2) *Device 2*, defined as systems possessing a dispersive stage with *β*_2(grating)_ = −890 ps^2^ m^−1^. This moderately large grating dispersion id designed to allow both spectral and temporal compression to occur in the device. In this system, Δ*Λ* = 6 nm over its length of 500 μm. (3) *Device 3*, defined as systems possessing a dispersive stage with *β*_2(grating)_ = −1,600 ps^2^ m^−1^, possesses the highest dispersion magnitude, designed to achieve stronger spectral compression. In this system, Δ*Λ* = 3.5 nm over its length of 500 μm. This device will be experimentally verified in the experimental section to give rise to stronger spectral compression compared to *Device 2*. The magnitude of *β*_2(grating)_ will be shown to impact the compression strength of the system.

**Fig. 3:**
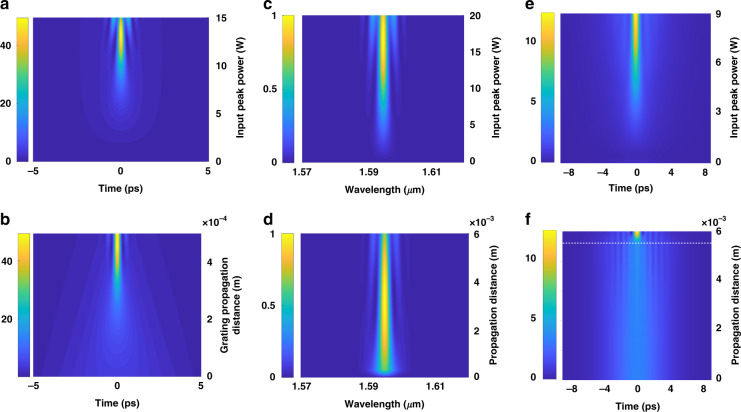
Numerically calculated pulse propagation dynamics for *Device1* and *Device 2*. **a** Temporal compression dynamics of *Device 1*, designed for high temporal compression, as a function of the input peak power for 5.8 ps input pulses. **b** Pulse temporal evolution as it propagates through the grating at a peak power of 13 W (pulse energy = 75.4 pJ). **c** Spectral compression dynamics as a function of input peak power for input pulses of 480 fs propagating in *Device 2*. Optimum spectral compression is achieved at a peak power of 18 W (pulse energy = 8.6 pJ). **d** Pulse spectral evolution as a function of propagation length for a peak power of 18.2 W (pulse energy = 8.7 pJ). **e** Output of the temporal pulse compressor (*Device 2*), as a function of input peak power for input pulses of 5.6 ps. Optimum temporal compression is achieved at a peak power of 6 W (pulse energy = 33.6 pJ). **f** Pulse temporal evolution in *Device 2* as a function of propagation length for a peak power of 9 W (pulse energy = 50.4 pJ). The dashed white line denotes where the USRN waveguide ends and the dispersive stage begins. Temporal compression proceeds quickly once pulses enter the dispersive stage. Plots (**a**, **b**) utilize *Device 1*, with *β*_2(grating)_ = −600 ps^2^ m^−1^, designed for high temporal compression. Plots (**c**–**f**) utilize Device 2 (*β*_2(grating)_ = −890 ps^2^ m^−1^), designed for reversibility in the spectro-temporal compression function. Color bars in **a**, **b**, **e**, **f** denote the output pulse peak power. Color bars in **c**, **d** denote the output pulse spectral intensity

*Device 1* (*β*_2(grating)_ = −600 ps^2^ m^−1^), designed for high temporal compression, is first studied. Fig. 3a shows the output of *Device 1* when the input peak power of 5.8 ps pulses is increased to 15 W. The extent of pulse compression is observed to increase as peak power is increased. New frequency components are generated through SPM, with the amount of temporal synchronization required to approach the transform limit decreasing as the peak power increases: this effect arises since a greater peak power generates a larger extent of spectral broadening, resulting in a smaller potential transform-limited pulse width. Fig. 3b further plots the pulse propagation dynamics within the grating section of *Device 1* at a fixed pulse peak power of 13 W, further elucidating the strong compression effects occurring in the dispersive stage. Next, we analyze *Device 2* which possesses *β*_2(grating)_ = −890 ps^2^ m^−1^, designed to allow both temporal and spectral compression to occur depending on which port is chosen as the input. Fig. 3d shows the spectral compression dynamics occurring wherein 480 fs pulses enter the dispersive stage first. The broad pulse spectrum narrows as pulses propagate through the USRN waveguide. SPM serves to continuously impart a frequency downshift to the temporally faster components and a frequency upshift to the temporally slower components. Consequently, it is apparent in Fig. 3d that the frequencies at the extremities of the pulse move closer to the center and result in an overall spectral compression effect. Next, the output of *Device 2* when operating as a spectral compressor is observed as a function of the input peak power (*P*_peak(in)_); from Fig. 3c, compression of the pulse spectrum is observed to become stronger as peak power is increased. The amount of frequency chirp acquired by the pulse scales with both nonlinear parameter (*γ*) and *P*_peak(in)_. At low powers, the extent of frequency down-/up-shifts imparted by SPM is insufficient to match the frequency-dependent delay introduced by the dispersive stage, and compression is not optimum. The pulse spectrum is observed to be narrowest at powers between 16 W–20 W, and the 3 dB bandwidth is reduced to 2.4 nm, corresponding to a spectral compression factor, $C_{\rm{s}} = \left( {\frac{{{\rm{Input}}\;{\rm{pulse}}\;3\,{\rm{dB}}\;{\rm{bandwidth}}}}{{{\rm{Output}}\;{\rm{pulse}}\;3\,{\rm{dB}}\;{\rm{bandwidth}}}}} \right)$ of 2.3 ×.

Next, the USRN waveguide is used as the input port for temporal compression for 5.6 ps pulses in *Device 2*. It is observed in Fig. 3e that temporal compression increases as peak power is increased. Optimum matching of the SPM-induced frequency chirp and that imparted by the fixed dispersion in the dispersive stage occurs at a power level of around 6 W. At this power level, the pulse experiences a temporal compression factor, $C_{\rm{T}} = \left( {\frac{{{\rm{Output}}\;{\rm{pulse}}\;{\rm{full}} - {\rm{width}} - {\rm{at}} - {\rm{half}} - {\rm{maximum}}}}{{{\rm{Input}}\;{\rm{pulse}}\;{\rm{full}} - {\rm{width}} - {\rm{at}} - {\rm{half}} - {\rm{maximum}}}}} \right)$of 4.3 ×; The pulse width exhibits a flattening at input peak power *P*_peak(in)_ > 6 W, indicating that pulses are maximally compressed. The degree of temporal compression induced by the dispersive grating can also be verified by the temporal pulse evolution through the compressor system. It is observed from Fig. 3f that as pulses propagate through the waveguide (*L* = 0 mm to 5.5 mm), negligible compression occurs. Significant temporal compression only occurs when pulses start to experience the grating (*L* = 5.5 mm–6 mm).

### Characterization of the USRN compressor designed for high temporal compression

Fabrication of the USRN compressor system is performed by first depositing 330 nm of USRN on a thermal SiO_2_ on Si substrate^43,44^. Details of the device fabrication, transmission, and dispersion properties of compressors incorporating gratings with Δ*Λ* = 3.5 nm, 6 nm and 8 nm giving rise to *β*_2(grating)_ = −1,600 ps^2^ m^−1^, −890 ps^2^ m^−1^, and −600 ps^2^ m^−1^, respectively are described in Supplementary Information 1. We first characterize *Device 1* (*β*_2_ = −600 ps^2^ m^−1^), designed for high temporal compression. 4.7 ps and 5.8 ps pulses derived from a fiber laser and centered at 1553.5 nm (repetition rate of 20 MHz) are used as input pulses in the temporal compression experiments. The chirp imposed by the two different phenomena are opposite in sign, and when the magnitudes are maximally matched, results in optimal temporal compression. The grating dispersion in this system is smaller than that in *Device 2*, possessing a value of −600 ps^2^ m^−1^ (Fig. S1c in Supplementary Information 1). This smaller magnitude of grating dispersion is instrumental to achieving high temporal compression: Temporally shorter pulses possess more spectral content. To generate strong temporal compression, greater nonlinear phase shift or more spectral broadening needs to occur in the USRN waveguide. Subsequently, a smaller magnitude of dispersion is needed to optimally rephase the larger extent of wavelengths in the compressed pulse spectrum, and in so forth appraoch optimal compression.

**Fig. 4:**
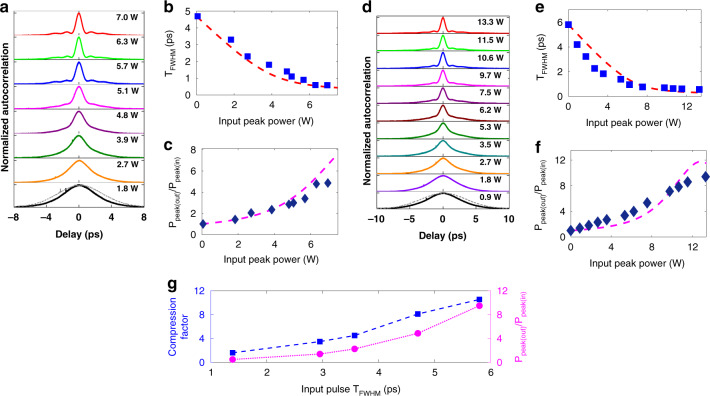
Temporal compression measurements using the USRN compressor (*Device 1*, *β*_2(grating)_ = −600 ps^2^ m^−1^) designed for high temporal compression. **a** Measured autocorrelation traces, **b** measured output pulse *T*_FWHM_ (blue squares) and **c** $\frac{{P_{{\rm{peak}}({\rm{out}})}}}{{P_{{\rm{peak}}({\rm{in}})}}}$ (blue diamonds) as a function input peak power of 4.7 ps pulses. **d** Measured autocorrelation traces, **e** measured output pulse *T*_FWHM_ (blue squares) and **f** $\frac{{P_{{\rm{peak}}({\rm{out}})}}}{{P_{{\rm{peak}}({\rm{in}})}}}$ (blue diamonds) as a function input peak power of 5.8 ps pulses. The numerically calculated *T*_FWHM_ and $\frac{{P_{peak(out)}}}{{P_{peak(in)}}}$ vs. input peak power are shown in the red dashed curves and magenta dashed curves, respectively. The dashed curve at (**e**) is extracted from Fig. 3a. A maximum temporal compression factor of 11× and peak power increase of 9.4× is achieved. **g** Experimentally measured compression factor and $\frac{{P_{{\rm{peak}}({\rm{out}})}}}{{P_{{\rm{peak}}({\rm{in}})}}}$ as a function of input pulse width

Fig. 4a shows the temporal compression traces as a function of input peak power when 4.7 ps pulses are launched into the pulse compression system. The temporal profile of the compressed pulses as a function of *P*_peak(in)_ is obtained using an autocorrelator. The output pulse full-width-at-half maximum (*T*_FWHM_) is achieved after applying the deconvolution factor (1.414 for Gaussian pulses) to the autocorrelation trace width. The minimum *T*_FWHM_ achieved for 4.7 ps input pulses is 0.58 ps when *P*_peak(in)_ = 7.0 W, corresponding to an 8.1× compression factor (Fig. 4b). We further characterize the ratio of the output pulse peak power to the input pulse peak power, $\frac{{P_{{\rm{peak}}({\rm{out}})}}}{{P_{{\rm{peak}}({\rm{in}})}}}$ to be 4.9× (Fig. 4c). Next, 5.8 ps pulses are used in the temporal compression experiments and the autocorrelation traces as a function of the input peak power of pulses are shown in Fig. 4d. As shown in Fig. 4e, when *P*_peak(in)_ = 13.3 W, a pulse width of 0.55 ps is achieved, corresponding to a compression factor of 11×. In addition, $\frac{{P_{{\rm{peak}}({\rm{out}})}}}{{P_{{\rm{peak}}({\rm{in}})}}}$ as a function of input pulse peak power is shown in Fig. 4f along with theoretically calculated values. The maximum value of $\frac{{P_{{\rm{peak}}({\rm{out}})}}}{{P_{{\rm{peak}}({\rm{in}})}}}$ experimentally achieved in this case is 9.4×, representing a large enhancement in the output peak power. In the temporal compression process, the peak power of the pulse is modified primarily by the pulse profile, including the presence and extent of pedestals, as well as linear losses of the device. Nonlinear losses from TPA and free-carrier effects have been shown to be absent in USRN^39,43^, and therefore have negligible impact on the evolution of the pulse shape, or in restricting the pulse compression dynamics. Three-photon absorption effects were also not observed at the power levels used in the experiments.

Fig. 4g further shows the experimentally measured compression factor and $\frac{{P_{{\rm{peak}}({\rm{out}})}}}{{P_{{\rm{peak}}({\rm{in}})}}}$ as a function of *T*_FWHM_. Both metrics are observed to increase as the pulse width is increased. This phenomenon is observed because sufficient nonlinear phase shift occurs for all the pulse widths, allowing the temporally longer input pulses to achieve a greater extent of compression compared to the shorter pulse width and given the fixed dispersion in the second stage of the device. The temporally compressed pulse profile as a function of input peak power for *T*_FWHM_ = 1.4 ps, 2.9 ps, and 3.6 ps is shown in Supplementary Information 2. It is also further observed from Fig. S2a, b that the severity of the pulse pedestals increases at the highest peak powers of this experiment. Beyond the optimal power level, the mismatch between the dispersive and nonlinear frequency chirp increases and results in the increasing amplitude of the pulse pedestals, a clear manifestation of a sub-optimal compression process.

### Characterization of the USRN compressor designed to allow both spectral and temporal compression

**Fig. 5:**
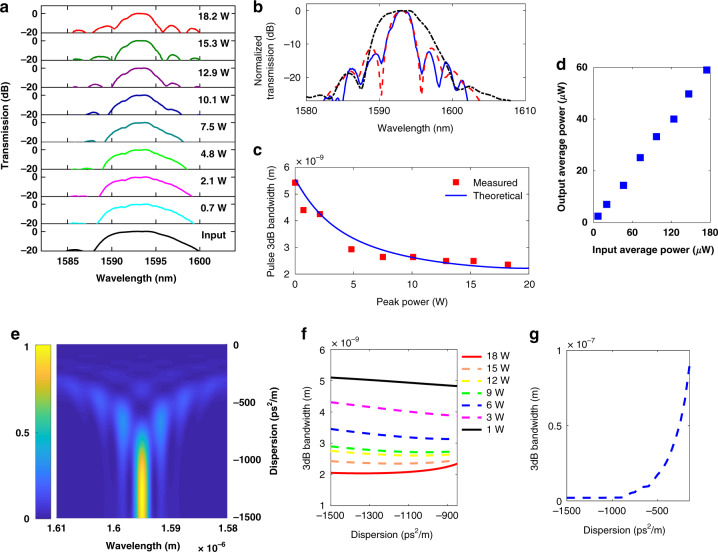
Experimental demonstration of spectral compression using *Device 2* (*β*_2(grating)_ = −890 ps^2^ m^−1^). **a** The measured output spectrum of the USRN compressor system when used as a spectral compressor as input peak power is increased. **b** The measured pulse spectrum for the source (black) and at the spectral compressor output when *P*_peak(in)_ = 18.2 W (blue) (pulse energy = 8.7 pJ). 2.3× spectral compression is achieved. The theoretical output pulse spectrum for *P*_peak(in)_ = 18.2 W is shown as the red dashed line. **c** The measured (red squares) and theoretical (blue line) 3 dB bandwidth (from Fig. 3c) of the output pulse as a function of input peak power. **d** Experimentally measured output average power vs. input average power. **e** Calculated output of the spectral compressor for a 480 fs input pulse with *P*_peak(in)_ = 18.2 W as a function of the grating dispersion. **f** Calculated 3 dB bandwidth of a spectrally compressed 480 fs input pulse as a function of dispersion magnitude in the dispersive stage, for different values of *P*_peak(in)_. **g** Calculated output 3 dB bandwidth for 480 fs input pulses as a function of dispersion magnitude in the dispersive stage, for *P*_peak(in)_ = 18.2 W

Next, we experimentally characterize *Device 2*, defined as systems possessing a dispersive stage with *β*_2(grating)_ = −890 ps^2^ m^−1^. This dispersion magnitude is designed to allow both spectral and temporal compression to occur to a moderate extent. For spectral compression measurements, an L-band fiber laser providing sub-picosecond, 480 fs pulses centered at 1593 nm with a repetition rate of 20 MHz are launched into compressor system with an operating wavelength centered at 1595 nm. Pulses are adjusted for quasi-TE polarization before coupling into the dispersive grating as the first stage followed by the USRN nanowire waveguide as the second stage. In this system, the anomalous dispersion in the grating first induces longer wavelengths to be delayed relative to the shorter wavelengths. Subsequently, the USRN waveguide equilibrates the frequency chirp by inducing a frequency downshift (upshift) in the temporally faster (slower) frequencies. The evolution of output spectra as a function of the input peak power is shown in Fig. 5a. Figure 5b compares the experimentally measured and numerically calculated pulse spectra when 2.3× spectral compression is achieved. Typically, SPM results in spectral broadening that increases as peak power is increased. The nonlinear phase acquired through the Kerr nonlinearity follows the equation, $\varphi _{{\rm{NL}}} = \gamma .P_{{\rm{peak}}({\rm{in}})}.L_{{\rm{eff}}}$. However, in observing the output of the spectral compressor, the output pulse bandwidth exhibits a different behavior, whereby an increase in *P*_peak(in)_ is accompanied by a pulse bandwidth reduction—this observation is a direct consequence of the time dependence of instantaneous frequency induced by the dispersive stage in the compressor system. At higher *P*_peak(in)_, multiple sidelobes arise, in similar fashion and physical origin as that observed in the temporal compressor. The spectral 3 dB bandwidth is observed to decrease as *P*_peak(in)_ is increased (Fig. 5c). The plateau observed in the extent of spectral compression arises from the fixed dispersion of the dispersive stage. A minimum 3 dB bandwidth of 2.8 nm is achieved, implying maximum spectral compression of 2.3× at the maximum *P*_peak(in)_ of 18.2 W. The theoretical 3 dB bandwidth as a function of the input peak power of the pulses is also plotted in Fig. 5c, showing good agreement with the measurements. The quality of the spectrally compressed pulse is also good, with the nearest pedestal located at the −13 dB level.

One of the key advantages of the spectral compression function over other pulse bandwidth reduction techniques such as bandpass filtering, is the preservation of pulse energy. Fig. 5d shows the measured output average power as a function of input average power. It is observed that the relationship is linear and fixed by the insertion losses of the device, implying that the reduction in the spectral bandwidth does not require truncation of the pulse energy.

The observed saturation in the extent of spectral compression arises mainly from the fixed dispersion in the dispersive stage. A larger magnitude of anomalous dispersion in the grating stage will allow stronger spectral compression to occur, provided sufficient nonlinear phase can be acquired in the USRN waveguide. Fig. 5e shows the calculated compressor output for 480 fs input pulses with *P*_peak(in)_ = 18.2 W as a function of *β*_2(grating)_. As the dispersion magnitude is decreased from −800 ps^2^ m^−1^ to −200 ps^2^ m^−1^, the pulse spectrum is observed to develop multiple lobes, indicating that the pulse evolution is increasingly dominated by the effects of SPM. It is observed from Fig. 5f that stronger spectral compression at *P*_peak(in)_ = 18.2 W is achieved when the dispersion magnitude is increased, but by a marginal amount of only 10%. This implies that the spectral compression achieved at *P*_peak(in)_ = 18.2 W is close to optimal. Fig. 5g shows the 3 dB bandwidth of the spectrally compressed pulse as a function of dispersion for *P*_peak(in)_ = 18.2 W in an expanded range. From the numerical calculation, it is further observed that when the dispersion in the dispersive stage drops beyond −800 ps^2^ m^−1^, spectral compression no longer occurs efficiently. As the dispersion magnitude decreases from −800 ps^2^ m^−1^ to −200 ps^2^ m^−1^, Fig. 5g shows that the output pulse width increases—a result of the dispersion being insufficient to balance the frequency shifts introduced by SPM in the second stage. These results confirm that both the presence of and magnitude of anomalous dispersion in the first stage are necessary elements for optimal spectral compression.

**Fig. 6:**
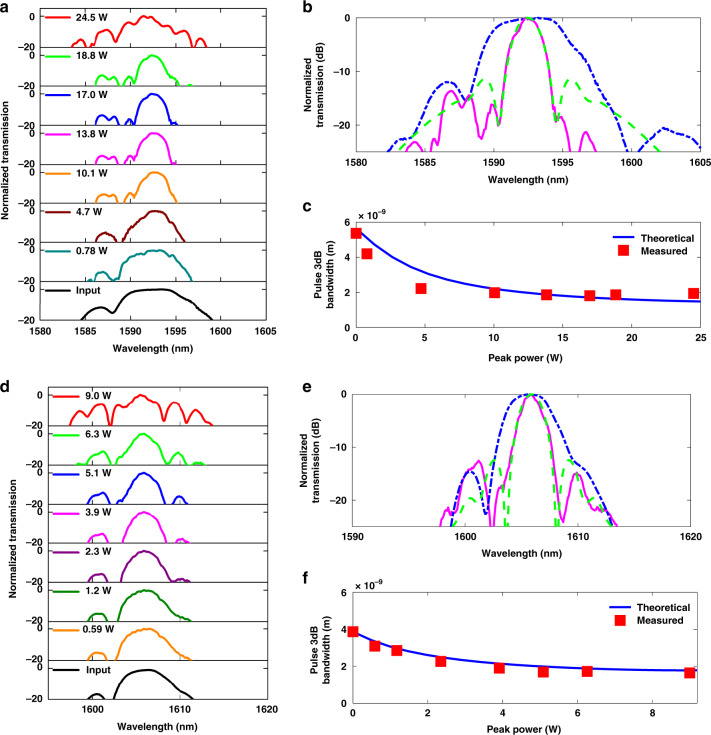
Impact of dispersion magnitude of the grating stage on spectral compression. **a** Output pulses as a function of input peak power for *T*_FWHM_ = 480 fs and *β*_2(grating)_ = −1,600 ps^2^ m^−1^. **b** Measured source (blue dashed line), compressed pulse (solid magenta line) and numerically compressed pulse (green dashed line) at optimal compression *P*_peak(in)_ = 14 W (pulse energy = 6.7 pJ). **c** Measured (red squares) and numerically calculated (blue solid line) spectrally compressed pulse width as a function of input peak power. **d** Output pulses as a function of input peak power for *T*_FWHM_ = 700 fs and *β*_2(grating)_ = −1,600 ps^2^ m^−1^. **e** Measured source (blue dashed line), compressed pulse (solid magenta line) and numerically compressed pulse (green dashed line) at optimal compression *P*_peak(in)_ = 5 W (pulse energy = 3.5 pJ). **f** Measured (red squares) and numerically calculated (blue solid line) spectrally compressed pulse width as a function of input peak power

Fig. 5g further shows that as the dispersion magnitude is increased beyond −1,500 ps^2^ m^−1^, spectral compression strengthens. This phenomenon occurs because pulses experiencing larger dispersion will temporally broaden more but cause a concomitant reduction in the pulse peak power. If sufficient nonlinear phase is acquired in the USRN waveguide stage, stronger spectral compression can take place when the magnitude of *β*_2(grating)_ is larger. This effect may also be observed when comparing the experimentally measured spectral compression of 480 fs pulses when a compressor system with a larger grating dispersion (*Device 3*; *β*_2(grating)_ = −1,600 ps^2^ m^−1^) is used. It is observed that the minimum 3 dB bandwidth achieved in this case is 1.8 nm at an input peak power of 14 W, equivalent to a spectral compression factor of 3.0× (Fig. 6a–c). It is further observed that as the input peak power increases beyond the optimum value, the sidelobes of the spectrally compressed pulses increase (Fig. 6a).

We further experimentally compare the compression of 700 fs pulses undergoing spectral compression in a two-stage system with *β*_2(grating)_ = −890 ps^2^ m^−1^ (Fig. S3a–c) and *β*_2(grating)_ = −1,600 ps^2^ m^−1^ (Fig. 6d–f). A stronger compression of 2.3× is achieved for *β*_2(grating)_ = −1,600 ps^2^ m^−1^, compared to 1.7× for *β*_2(grating)_ = −890 ps^2^ m^−1^, further confirming the relationship between spectral compression strength and magnitude of *β*_2(grating)_. This phenomenon may also be inferred when comparing the spectral compression occurring for different pulse widths (480 fs and 700 fs) for a fixed value of *β*_2(grating)_. For a fixed grating dispersion, temporally longer source pulses will undergo less temporal broadening in the dispersive stage, and consequently undergo weaker spectral compression. In Table 1, it is observed that the spectral compression is stronger for 480 fs pulses vs. 700 fs pulses, for both *β*_2(grating)_ = −890 ps^2^ m^−1^ and −1,600 ps^2^ m^−1^. We note further that the delicate interplay between dispersion, nonlinearity, and pulse peak power needs to be considered: if the pulses are temporally broadened too much in the dispersive stage, the peak power of the pulse entering the USRN waveguide stage will be low, making it more difficult to acquire sufficient nonlinear phase. Table 1 shows the achieved spectral compression for different pulse widths and *β*_2(grating)_.

**Table 1:** Experimentally measured spectral compression factor as a function of *β*_2(grating)_ and *T*_FWHM_

*β*_2(grating)_ (ps^2^/m)	*T*_FWHM_ (fs)	Compression Factor
−890	480	2.3 ×
−1600	480	3.0 ×
−890	700	1.7 ×
−1600	700	2.3 ×

A larger magnitude of *β*_2(grating)_ is experimentally confirmed to generate stronger compression.

**Fig. 7:**
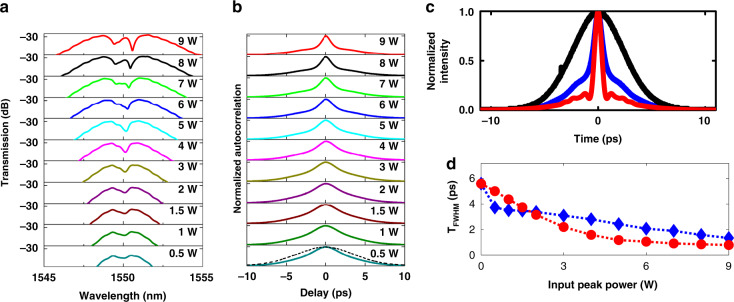
Experimental demonstration of temporal compression using *Device* 2 (*β*_2(grating)_ = −890 ps^2^ m^−1^). The measured (**a**) output spectra (**b**) and temporal traces of the USRN compressor. The dashed line in (**b**) shows the temporal trace for input laser. **c** The measured (blue) and theoretical temporal pulses (red) is plotted along with input pulse (black) at *P*_peak(in)_ = 9 W (pulse energy = 50.4 pJ). **d** Measured pulse width (blue diamonds) as a function of *P*_peak(in)_ fitted by theory (red circles). The theory is extracted from Fig. 3e

Next, temporal compression experiments are performed in *Device 2*, by launching pulses into the nonlinear stage first. 5.6 ps pulses centered at 1550 nm at a repetition rate of 20 MHz are derived from a fiber laser. The result is shown in Fig. 7. The autocorrelation trace as a function of input peak power is shown in Fig. 7b. It is observed that the autocorrelation trace width decreases monotonically as *P*_peak(in)_ is increased to 9 W. When *P*_peak(in)_ = 9 W, autocorrelation trace width is measured to be 2.2 ps, equivalent to *T*_FWHM_ of 1.4 ps and corresponding to a temporal compression factor of 4.3×. For completeness, the spectral evolution is shown in Fig. 7a. The spectral content of the pulse increases as a function of input peak power, consistent with the scaling of SPM-induced nonlinear phase acquisition with peak power. It is further observed that the evolution of the pulse spectrum is highly symmetric, consistent with negligible free-carrier effects in the USRN platform.

A comparison of the theoretical and experimental pulse traces at *P*_peak(in)_ = 9 W is shown in Fig. 7c. Experimental pulse traces (blue solid lines) are plotted after performing a sech^2^ deconvolution on the autocorrelation traces. It is observed that the main lobe of the experimental pulse (blue solid line) trace agrees well with the theory (red solid line), as shown for the plot at the maximum *P*_peak(in)_. The presence of pulse pedestals in the experimental trace implies that not all the input pulse energy contributes to the main lobe of the compressed pulse. Pulse pedestals arise as a result of incomplete matching of the frequency chirp profiles (magnitude and shape) derived from the nonlinear and dispersive stages. In particular, the nonlinear parts of the SPM-induced frequency chirp (blue color in the curve of Fig. 2a (inset)) have a considerable contribution to the formation of pulse pedestals in the output pulses at higher peak powers. Fig. 7d further shows the theoretical and experimental pulse FWHM as a function of *P*_peak(in)_. *T*_FWHM_ is observed to decrease monotonically to its minimum value of 1.4 ps at an input peak power of 9 W. Physically, the dispersive stage supports a fixed compressed pulse width that would be closest to the transform limit. As the SPM-induced frequency chirp increases beyond this, the rate at which compression increases with *P*_peak(in)_ decreases and pulse pedestals emerge.

Further insight regarding the role of the designed values of *β*_2(grating)_ can be obtained by comparing the temporal compression results obtained in *Devices 1* and *2*. *Device 1* possesses a smaller grating dispersion, and can therefore support a compressed pulse with a potentially shorter temporal width, provided the requisite frequency content is generated in the nonlinear stage: At an input peak power of ~9 W and similar starting pulse widths, *Devices 1* and *2* generate compressed pulses with *T*_FWHM_ = 0.69 ps (8.4× compression) and 1.4 ps (4.3× compression) respectively. The design of the dispersive stage is, therefore, a critical factor in the temporal compression process, and may be optimized for a desired compressed pulse width given specific starting pulse conditions. In addition, a control experiment that compares the temporal output of the system when a compressor system with a grating without any engineered dispersion is provided in Supplementary Information 4. In this case, it is observed that no temporal compression occurs, further highlighting the importance of the dispersive stage in the compression process.

## Discussion

In this work, a unique capability of spectral or temporal compression achievable using the same integrated system has been demonstrated for the first time. Of note, the design flexibility afforded by decoupling the nonlinear and dispersive stages allows pulses of various widths to be manipulated. We experimentally achieved a maximum temporal compression factor of 11×, accompanied by an increase in pulse peak power by 9.4× at a low peak power of 13.3 W. These represent one of the best performance demonstrated in on-chip pulse compression systems. This work also represents the first demonstration of efficient optical spectral compression on a CMOS-chip and may spawn various new approaches toward ultrafast pulse manipulation on integrated platforms. A maximum spectral compression factor of 3.0× is achieved at a peak power of 14 W.

Within the domain of temporal compression on a chip, soliton-effect compression has been shown to provide good performance^8,10–12,15–17,45^. Pulse manipulations relying on solitons depend critically on the dispersive properties on the waveguide. Since the dispersive length = *T*_0_^2^/|*β*_2_|, wider pulse widths entail long dispersive lengths, making it more difficult to achieve strong compression at low powers. Solitons supported by periodic structures are better options for the compression of wider pulses, providing three orders of magnitude larger dispersion compared to photonic waveguides, and equivalently shorter dispersive lengths^10,12,15–17,45,46^. Demonstrations of CMOS-chip based temporal compression have been implemented on platforms ranging from Si (high Kerr nonlinearity, low nonlinear figure of merit), to Si_3_N_4_ (TPA free, smaller Kerr nonlinearity). Because of the fundamental requirement to efficiently generate new frequencies during the temporal compression process, tradeoffs between power efficiency, operating power, and compression strength exist in these demonstrations. These tradeoffs are further highlighted in Fig. 1b which compares the compression factors achieved, $\frac{{P_{{\rm{peak}}({\rm{out}})}}}{{P_{{\rm{peak}}({\rm{in}})}}}$ and peak powers required for various CMOS-chip based temporal compression demonstrations. For example, 2.3× soliton-effect compression was demonstrated in silicon photonic crystal waveguides using *P*_peak(in)_ = 2.4 W^12^, whereas 5× compression was demonstrated in Si_3_N_4_ waveguides using considerably higher peak powers ~3.2 kW^16^. Since USRN has a large Kerr nonlinearity and governed by three-photon (rather than two-photon) loss mechanisms at 1550 nm^39^, relatively small powers are required to initiate compression^40,41^, while importantly preserving the pulse energy. The temporal compression performance of the USRN system is further plotted in Fig. 1b, showing its superior performance for both compression factor and, $\frac{{P_{{\rm{peak}}({\rm{out}})}}}{{P_{{\rm{peak}}({\rm{in}})}}}$. The advantages of decoupling the dispersive and nonlinear stages in the compression system are also clear from the significantly stronger temporal compression achieved in *Device 1* vs. *Device 2*, where the grating dispersion was specifically tailored for high temporal compression. Similarly, stronger spectral compression is achieved in *Device 3* vs. *Device 2*, where the dispersion is tailored specifically for spectral compression. In addition, the dispersive stage has favorable group delay dispersion properties, exhibiting a linear profile with minimal group delay ripple. Consequently, the profile of the dispersion and SPM-induced frequency chirp is well matched. We note that AlGaAs, on which octave spanning coherent supercontinuum^47^ and frequency comb generation^48^ have been demonstrated, possesses some similar advantageous features as USRN, including high nonlinearity and TPA-free behavior, and could also be well suited for the implementation of nonlinear pulse compression schemes.

It was previously reported in Ref. ^9^. that 7.0× temporal compression in a silicon temporal pulse compressor was achieved with *P*_peak(in)_ = 10 W, with the increase in the pulse peak power being limited to 1.8×; spectral compression was not reported. It is, therefore, of interest to compare the performance of the USRN compressor system vs. the Si temporal pulse compressor. Table 2 compares various performance metrics of the USRN compressor system vs. the Si pulse compressor. It is clear that the USRN compressor performs better in the various metrics. In the Si system, the temporal compression and increase in peak power was reported to plateau quickly, reportedly from competing two-photon and free-carrier effects, and severely limiting the achievable $\frac{{P_{{\rm{peak}}({\rm{out}})}}}{{P_{{\rm{peak}}({\rm{in}})}}}$. Increases in input peak power result in higher nonlinear absorption, and result in diminishing performance. In the current work, a maximum temporal compression factor of 11× is achieved, as well as an increase in the pulse peak power by 9.4× at a low peak power of 13.3 W. These compression metrics are significantly better than that demonstrated in the Si system. Furthermore, a comparison of the pulse quality at the highest compression factors reveals that the compressed pulses in the USRN system have much lower pulse pedestals.

**Table 2:** Comparison between USRN compressor (this work) and ref. ^9^

	USRN	Si
Temporal compression factor (×)	11	7.0
Peak power increase (×)	9.4	1.8
Spectral compression factor (×)^a^	3.0	1.09
Efficiency at high repetition rates	High	Low
Nonlinear losses at 1550 nm	No	Yes

^a^The spectral compression factor for Si is based on numerical calculations in Supplementary Information 5.

Additional numerical studies comparing the performance of the USRN and Si systems are provided in Supplementary Information 5. Firstly, the performance of the system as a function of pulse repetition rate is studied. The simulations reveal that the presence of free-carriers in Si places a limit on the usable pulse repetition rates. Comparing the performance of the dual-stage system when implemented in USRN and Si, it is shown that the temporal compression process deteriorates at higher repetition rates for Si as a result of the finite free-carrier lifetime, which is on the order of nanoseconds. At a repetition rate of 10 GHz, the compression efficiency in Si is halved compared to that at the single pulse regime (Fig. S5b). This deterioration in compression efficiency arises because free carriers excited by a pulse do not have time to dissipate before the next pulse arrives, leading to a large buildup of free carriers. Conversely, the compression process proceeds uncompromised in USRN. Consequently, silicon is not ideal for nonlinear compression when repetition rates approach 10 GHz or higher. Though it may be argued that the free-carrier dispersion effect may confer additional phase shifts beneficial to the compression process, it has been previously shown through limitations in the compression factor^12^ that the efficiency of the compression process is significantly undermined by free carriers and the two-photon transitions that generate them. Furthermore, the peak power increase is small compared to that achieved in USRN (1.8× in Si vs. 9.4× in USRN), due to high nonlinear losses in Si.

It is further shown that spectral compression in the Si system is weak (Fig. S5a), allowing only a negligible, 8% decrease in the 3 dB bandwidth of a 480 fs pulse. When implemented as a spectral compressor, pulses will enter the dispersive stage first, and therefore encounter a reduction in the pulse peak power at the first stage, both from any losses present in the dispersive stage and the dispersion induced temporal broadening. In silicon, the pulses will experience significant two-photon and free-carrier losses at the dispersive stage, exacerbating the attenuation of the pulse power needed for nonlinear phase acquisition in the second stage^9,49,50^.

An alternative method of reducing the spectral bandwidth of a pulse involves spectral filtering. However, this method requires truncation of the pulse spectrum, inevitably leading to loss in the pulse energy. The demonstrated method of reducing pulse spectral bandwidth retains all the pulse energy and generates spectral compression by retiming the frequency components, followed by enabling frequency shifts that serve to reduce the spectral bandwidth. When combined with the negligible nonlinear loss in the USRN platform, this spectral compression scheme provides a highly efficient method for bandwidth reduction of optical pulses. The spectrally compressed pulses demonstrated in this work have pedestals that are relatively low, −13 dB lower than the main lobe. The origin of the pedestals in the spectrally compressed pulses is the non-perfect match between the dispersive and nonlinear frequency chirp^22,50^. The level of the pedestals may be further decreased by introducing normal dispersion in the USRN waveguide, which serves to linearize the nonlinear frequency chirp. Fig. S6a in Supplementary Information 6 shows that the level of the spectrally compressed pulse pedestals decreases by 2.5 dB as the dispersion of the USRN waveguide is increased from −0.2 ps^2^ m^−1^ to 40 ps^2^ m^−1^. A nonlinear stage with large magnitude of normal dispersion has a similar positive effect on reducing the pulse pedestals of the temporally compressed pulse pedestals (Fig. S6b). This effect arises because the normal dispersion serves to linearize the frequency chirp introduced by the nonlinear stage, providing a better match with the dispersive chirp.

We have demonstrated a pulse compressor system on a CMOS-compatible chip, capable of high temporal and spectral compression. Strong temporal compression (11×) of 5.8 ps pulses to 0.55 ps is demonstrated on a CMOS-compatible USRN chip at a low pulse peak power of 13.3 W. This compression process is accompanied by a large peak power increase in the output pulses of 9.4×. Furthermore, 3.0× spectral compression of 480 fs pulses is demonstrated. This work further reflects the ability for backend CMOS-compatible USRN to efficiently implement sophisticated optical signal processes. The results represent a new paradigm of ultrafast pulse waveform manipulation, providing direct, tailored control of the pulse spectrum and temporal profile using the same integrated system.

## Materials and methods

### Fabrication

Fabrication of the USRN system is performed by first depositing the 330 nm of USRN on a 3 µm thermal oxide layer on silicon substrate using inductively coupled plasma chemical deposition at a low temperature of 250 °C implying back-end CMOS compatibility. The device was patterned using electron-beam lithography, reactive ion etching and deposition of 2 μm-thick SiO_2_ overcladding using atomic layer deposition and plasma enhanced chemical vapor deposition. The fabricated waveguides have a propagation loss of 3 dB/cm, the dispersive grating loss for *β*_2(grating)_ = −600 ps^2^ m^−1^ and −890 ps^2^ m^−1^ is 3 dB. The dispersive grating loss for *β*_2(grating)_ = −1,600 ps^2^ m^−1^ is ~1 dB. Inverse tapers are used to facilitate fiber-waveguide coupling. The coupling loss at each fiber-waveguide facet is 6 dB.

### Numerical calculations

Pulse propagation dynamics are analyzed using the nonlinear Schrödinger equation (NLSE),

$$\frac{{\partial A}}{{\partial z}} = - \frac{\alpha }{2}A - i\frac{{\beta _2}}{2}\frac{{\partial ^2A}}{{\partial T^2}} + i\gamma \left| A \right|^2A,$$

where *α* is the linear loss coefficient of 0.69 cm^−1^, *A* and *ω*_0_ are the slow varying pulse envelope and carrier frequency, respectively. The temporal coordinate, *T* exists in a moving frame due to the removal of the temporal walk-off induced by the group velocity^42^. The linear and nonlinear refractive indices of USRN are 3.1 and 2.8 × 10^−13^ cm^2^ W^−1^, respectively^39,43^ The effective mode area of the USRN waveguide is calculated to be 0.26 µm^2^ resulting in a nonlinear parameter (*γ*) of 440 W^−1^ m^−1^. The calculated second order dispersion, *β*_2_ for the waveguide = −0.17 ps^2^ m^−1^ at wavelength of 1550 nm. In the simulations, the dispersion in the dispersive stage, *β*_2(grating)_ = −600 ps^2^ m^−1^ and −890 ps^2^ m^−1^ for *Device 1* and *Device 2*, respectively. For modeling of the silicon system, parameters from ref. ^9^ are used.

### Spectral and temporal measurements

Tapered lensed fibers were used to couple light into and out of the dual-stage system. The pulses used for the temporal compression experiments originated from a mode locked fiber laser (MLFL) operating at a repetition rate of 20 MHz. In the experiments, the 5.6 ps pulses (sech^2^) used in the experiments were derived from the MLFL. A tunable bandpass filter (resulting in Gaussian shaped pulses) was used to derive the 1.4 ps, 2.9 ps, 3.6 ps, 4.7 ps, and 5.8 ps pulses from the pulses originating from the fiber laser, prior to amplification using an erbium-doped fiber amplifier to achieve the peak powers used in the experiments. The spectral output was measured using an optical spectrum analyzer whereas the temporal traces were retrieved using an autocorrelator. To increase the signal to noise ratio, 256 autocorrelation traces are averaged for each measured temporal pulse profile.

The 480 fs and 600 fs pulses (sech^2^) used for spectral compression experiments were derived from a mode locked fiber laser at a repetition rate of 20 MHz with an integrated wavelength converter emitting pulses in the L-band. The spectral measurements for spectral compression were performed using an optical spectrum analyzer. Measurements of the output average power of the spectral compressor were performed using a power meter, normalized to the fiber-waveguide coupling losses.

### Calculation of ratio of output pulse peak power to input pulse peak power

The calculation of $\frac{{P_{{\rm{peak}}({\rm{out}})}}}{{P_{{\rm{peak}}({\rm{in}})}}}$ is performed using a method similar to that used in refs. ^9^ and ^13^

$$\frac{{P_{{\rm{peak}}({\rm{out}})}}}{{P_{{\rm{peak}}({\rm{in}})}}} = \frac{{(P_{{\rm{ave}}}.f)}}{{P_{{\rm{peak}}(in)}.R_{\rm{p}}.\mathop {\int }\nolimits_{ - \infty }^\infty P(t)dt}},$$

where *P*_ave_ is the average output power from the compressor system measured using a power meter, normalized to the device insertion and coupling losses. *f* is the estimated energy fraction within the main lobe of the pulse and *P*_peak(in)_ is the pulse peak power coupled into the compressor system. *R*_P_ is the pulse repetition rate, *P*(*t*) is the normalized pulse intensity assuming a Gaussian profile with *T*_FWHM_ measured from the autocorrelation traces.

## Supplementary information

Supplementary Information
