# Supplementary material for: High spectro-temporal compression on a nonlinear CMOS-chip
Source: Light Sci Appl. 2021 Jun 18;10:130. doi: 10.1038/s41377-021-00572-z (PMC8211677; doi:10.1038/s41377-021-00572-z)
Supplement: Supplementary file 1 — Supplementary Information [file 41377_2021_572_MOESM1_ESM.docx]

**High spectro-temporal compression on a nonlinear CMOS-chip**

**Supplementary Information**

Ju Won Choi^1^, Ezgi Sahin^1^, Byoung-Uk Sohn^1^, George F. R. Chen^1^, Doris K. T. Ng^2^, A. M. Agarwal^3,4^, L. C. Kimerling^4,5^ & Dawn T. H. Tan^1,*^

^1^Photonics Devices and System Group, SUTD-MIT International Design Center, Singapore University of Technology and Design, Singapore 487372, Singapore

^2^Institute of Microelectronics, A*STAR (Agency for Science, Technology and Research), 2 Fusionopolis Way, #08-02, Innovis Tower, Singapore 138634, Singapore

^3^Microphotonics Center, Massachusetts Institute of Technology, 77 Massachusetts Avenue, Cambridge, Massachusetts 02139, USA

^4^Materials Research Laboratory, Massachusetts Institute of Technology, 77 Massachusetts Avenue, Cambridge, Massachusetts 02139, USA

^5^Department of Materials Science and Engineering, Massachusetts Institute of Technology, 77 Massachusetts Avenue, Cambridge, Massachusetts 02139, USA

^*^Corresponding author: dawntan@mit.edu

**SUPPLEMENTARY INFORMATION 1: CHARACTERIZATION OF DISPERSIVE STAGES**

The transmission characteristics of a grating with Δ*Λ* = 6 nm is first characterized. The measured transmission is shown as the blue curve in Fig. S1a. The corresponding group delay profile is shown in Fig. S1b and measured two ways: (i) Interferometrically (red diamonds) and (ii) using time of flight measurements with a dispersion analyzer (magenta circles). In the interferometric dispersion measurement, a Fabry Perot cavity between the cleaved waveguide facet and the reflection point of the light in the grating exists and results in oscillations in the transmission spectrum. The optical path length traveled by each wavelength component follows the relation, *L*(*λ*)=*λ*^2^/(2*n*_g_*Δλ*), where *λ* is the wavelength, *n*_g_ is the group index of the waveguide and *Δλ* is the Fabry Perot oscillation period. The group delay as a function of wavelength, *τ*(*λ*) = 2*n*_g_*L*(*λ*). A second measurement method utilized a dispersion analyzer that leverages time of flight measurements to extract the group delay as a function of wavelength. The group delay spectrum measured using both methods agree well. For both methods, the slope of the *τ*(*λ*) plot as a function of *λ* is the extracted dispersion (Fig. S1b).

The group delay dispersion for the grating with Δ*Λ* = 6 nm is extracted using the magnitude of the slope of group delay vs. wavelength to be 0.35 ps nm^-1^, equivalent to a GVD of -890 ps^2^ m^-1^. In contrast, the calculated GVD of the USRN waveguide in the nonlinear stage is -0.17 ps^2^ m^-1^, a value that is significantly smaller compared to that of the dispersive stage; any dispersion induced frequency chirp is therefore dominated by the dispersive stage, with negligible contributions from the nonlinear stage. The grating insertion loss is ~3 dB. This loss is dominated by incomplete coupling of the incident optical field and may be resolved by increasing the coupling strength. Figures S1c and S1d show the transmission and group delay properties of gratings with Δ*Λ* = 8 nm and 3.5 nm respectively, generating in interferometrically extracted dispersion of -600 ps^2^ m^-1^ and -1600 ps^2^ m^-1^ respectively. The grating with Δ*Λ* = 3.5 nm has the lowest insertion loss of ~1 dB, due to the greater overall coupling associated with the smaller grating chirp.

­

**Figure S1.** Optical characterization of gratings used in the compression systems. **a** Measured transmission spectrum for the dispersive stage with Δ*Λ* = 6 nm showing a 3 dB bandwidth of 25 nm. **b** Measured group delay characteristics of a dispersive stage with Δ*Λ* = 6 nm. The group delay measured interferometrically is represented by the red diamonds. Group delay measured using time of flight in a dispersion analyzer is shown as the magenta circles. The black dotted line is the linear fit to the group delay vs. wavelength profile. The dispersion extracted from the group delay vs. wavelength plot is -890 ps^2^ m^-1^, the value of dispersion that allows both spectral and temporal compression to take place. For completeness, the measured transmission is shown in the same plot as the blue curve. **c** Characterization of grating with Δ*Λ* = 8 nm used for the 8.1 × and 11 × temporal compression experiments. The transmission and group delay spectrum are plotted in blue and magenta respectively. The black dotted line is the linear fit to the group delay spectrum. The dispersion extracted from the group delay vs. wavelength plot is -600 ps^2^ m^-1^. **d** Characterization of grating with Δ*Λ* = 3.5 nm, used for the 3.0 × spectral compression experiments. The dispersion extracted from the group delay vs. wavelength plot is -1600 ps^2^ m^-1^. The transmission and group delay spectrum are plotted in blue and magenta respectively. The black dotted line is the linear fit to the group delay spectrum.

**SUPPLEMENTARY INFORMATION 2: ADDITIONAL TEMPORAL COMPRESSION MEASUREMENTS**


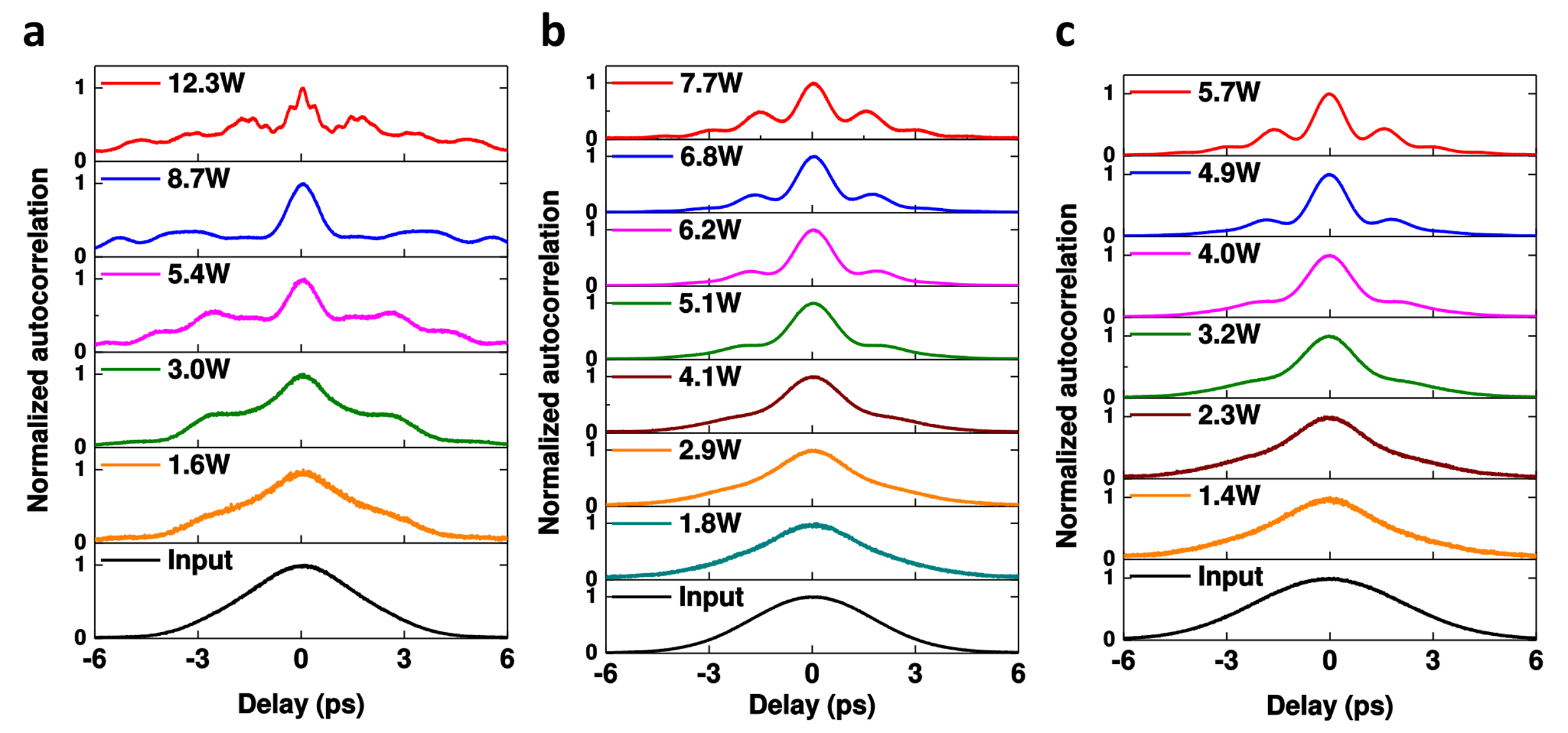


**Figure S2.** Additional temporal compression measurements for 1.4 ps, 2.9 ps and 3.6 ps input pulses. Normalized autocorrelation of the **a** 1.4 ps, **b** 2.9 ps and **c** 3.6 ps pulses as a function of input peak power when propagating through the temporal compressor system with *β*_2(grating)_ = -600 ps^2^ m^-1^.

Additional temporal compression measurements using Device 1 (*β*_2(grating)_ = -600 ps^2^ m^-1^) were performed in order to investigate the evolution of the achievable compression factor as a function of input pulse *T*_FWHM_. Figure S2a, S2b and S2c respectively show the output pulse traces as a function of the input peak power of the pulses for 1.4 ps, 2.9 ps and 3.6 ps pulses. The severity of the pulse pedestals is observed to increase at the highest peak powers. In these cases, the mismatch of the nonlinear and dispersive chirp becomes large, leading to a compressed pulse profile where more energy resides outside of the main lobe.

**SUPPLEMENTARY INFORMATION 3: ADDITIONAL SPECTRAL COMPRESSION MEASUREMENTS**

**Figure S3.** Additional spectral compression measurements. **a** Spectral compression of 700 fs pulses (*β*_2(grating)_ = -890 ps^2^ m^-1^) as a function of input peak power. **b** The normalized transmission for the input pulse (blue dashed) measured (solid magenta) and calculated (green dashed) output pulse for *P*_peak(in)_ = 11.7 W (pulse energy = 8.2 W). **c** The calculated (blue line) and experimentally measured (red squares) output pulse 3 dB bandwidth as a function of input peak power.

Figure S3 shows the spectral compression occurring in a two-stage system with (*β*_2(grating)_ = -890 ps^2^ m^-1^). The evolution of the compressed pulse as a function of input peak power (Fig. S3a) show that the sidelobes increase at higher powers. The calculated and measured compressed pulse at *P*_peak(in)_ = 11.7 W is also shown in Fig. S3b, as well as the theoretical and measured compressed pulse 3dB bandwidth as a function of *P*_peak(in)_ (Fig. S3c).

**SUPPLEMENTARY INFORMATION 4: CONTROL EXPERIMENT**

A control experiment where 9 ps pulses at a peak power of 3.6 W are launched into the temporal compressor system but using a grating without any engineered group delay dispersion is also shown in Fig. S4. Fig. S4a shows the transmission spectrum of the system where the dispersive stage does not possess any engineered group delay dispersion. The bandwidth of the system is reduced because of the absence of any engineered group delay dispersion. Fig. S4b shows the increase in the spectral bandwidth of the 9 ps pulse after propagating through the system. However, the increase in spectral content is not accompanied by any reduction in temporal width. Fig. S4c shows the original 9 ps pulse and the pulse profile at the output of the system. It is observed that pulses do not experience temporal compression, and instead the pulses exhibit a marginal broadening. Conversely, 9 ps pulses propagating through the *Device 2* (*β*_2(grating)_ = -890 ps^2^ m^-1^) experiences temporal compression (red solid line in Fig. S4c). These results confirm the criticality of the dispersive stage in the compression process, particularly to equilibrate any frequency chirp originating from the nonlinear stage. The spectral and temporal traces of 9 ps input pulses are shown in Figure S4d and S4e respectively, as peak power increases.

**Figure S4**. Control experiment with a compressor system with zero engineered dispersion in the dispersive stage. **a** Measured transmission spectrum of a dispersive grating without any engineered dispersion. The measured 3 dB bandwidth is 6 nm. **b** Measured spectra of 9 ps input pulse (red dashed line) and output pulse from the system with zero engineered dispersion, at a peak power of 3.7 W (pulse energy = 33.3 pJ). **c** Measured normalized intensity (deconvolved autocorrelation traces) of the input (black solid line) and output (blue solid line) pulses when launched into the compressor system with a dispersive stage with zero engineered dispersion. showing negligible temporal compression. The output pulse when launched into the temporal compressor system with (*Device 2* with *β*_2(grating)_ = -890 ps^2^ m^-1^). Temporal compression is observed in this case. The inset shows the theoretical input (black dashed line) and output (blue dashed line), and measured output (red dashed line) when the pulse is launched into *Device 2* (*β*_2(grating)_ = -890 ps^2^ m^-1^). The measured **d** output spectra and **e** temporal traces as a function of peak power using a 9 ps input pulse in *Device 2* (*β*_2(grating)_ = -890 ps^2^ m^-1^).

**SUPPLEMENTARY INFORMATION 5: NUMERICAL ANALYSIS OF THE USRN COMPRESSOR PERFORMANCE VS. SILICON COMPRESSOR**

Both USRN and silicon have a high linear and nonlinear refractive index. This facilitates efficient self-phase modulation necessary for the nonlinear process in the compressor performance. Unlike USRN, silicon has non-negligible two-photon and free-carrier absorption at wavelengths below 2.1 μm. Extensive numerical modeling is performed to further showcase the relative merits of USRN vs. silicon compressors.

The performance of the Si-based compressor is first evaluated when operated as a spectral compressor. The parameters for the silicon device are obtained from Ref. S1. 480 fs Gaussian pulses are launched into the dispersive stage first prior to their propagation in the nonlinear silicon nanowire waveguide. It is observed from Fig. S5a that there is a marginal decrease in the 3 dB bandwidth as the pulse power is increased to 20 W (7.3 nm to 6.7 nm). The corresponding compression factor achieved at the highest power of 20 W is 1.09 ×, implying that the pulse spectrum has been negligibly compressed.

We further studied the impact of repetition rate on the compression efficiency when implemented in USRN and silicon. It has previously been reported that at telecommunications wavelengths, silicon’s free-carrier lifetime in high confinement waveguide geometries is on the order of nanoseconds [S2, S3]. USRN on the other hand does not have two-photon or free-carrier effects at 1550nm [S4, S5]. Therefore, we study the performance of the USRN compressor when used for temporal compression, as a function of pulse repetition rate. The performance is compared with silicon compressors. In these simulations, the free carrier lifetime of silicon used is 1 ns and the pulse shape is assumed to be Gaussian. Figure S5b shows the ratio, *T*_SP_/*T*_RP_ for the USRN (*T*_FWHM_ = 5.8 ps, *P*_peak(in)_ = 13 W) and silicon compressor (*T*_FWHM_ = 7 ps, *P*_peak(in)_ = 10 W), when operated for temporal compression. *T*_SP_ denotes the compressed pulse width in the single pulse regime and *T*_RP_ denotes the compressed pulse width in the multi-pulse regime at a fixed repetition rate, *R*_P_. It is observed from Fig. S5b that the efficiency of the temporal compression process increasingly deteriorates in the multi-pulse regime when the repetition rate increases from 0.5 GHz to 28 GHz. At 10 GHz and 28 GHz, the compressed pulse width is 2 × and 3 × wider than in the single pulse regime. This deterioration in the compression process arises because at higher repetition rates, free-carriers generated by a pulse do not have sufficient time to dissipate before the next pulse arrives, causing a large buildup of free carriers.

Conversely, the compression process is observed to proceed uncompromised in USRN. In USRN, the ratio of the compressed pulse width in the single and multi-pulse regime continues to be 1. This is shown in Fig. S5b where *T*_SP_/*T*_RP_ = 1 for *R*_P_ up to 28 GHz. Consequently, USRN is a much better CMOS platform compared to silicon for implementation of the pulse compression system when repetition rates approach 10 GHz or higher. Though it may be argued that the free-carrier dispersion effect may confer additional phase shifts beneficial to the compression process, it has been previously shown through limitations in the compression factor [S6] that the efficiency of the compression process is significantly undermined by free carriers and the two-photon transitions that generate them. Furthermore, the peak power increase is small compared to that achieved in USRN (1.8 × in Si vs. 9.4 × in the USRN compressor), due to high nonlinear losses in Si.

**Figure S5.** Numerical analysis of the USRN compressor performance vs. the silicon compressor. **a** Performance of a silicon compressor when operated for spectral compression. The 3 dB bandwidth (blue) and compression factor (orange) are plotted as a function of the peak power of a 480 fs pulse (Gaussian). **b** *T*_SP_/*T*_RP_ as a function of pulse repetition rate in a USRN and silicon compressor when used as a temporal compressor.

**SUPPLEMENTARY INFORMATION 6: NUMERICAL ANALYSIS OF THE COMPRESSED PULSE PROFILE**

At the highest compression factor of 2.3 ×, the spectrally compressed pulse has pedestals at the -13 dB level. This is a result of the non-ideal matching of the frequency chirp generated in the dispersive stage and the nonlinear stage. The SPM-induced frequency chirp profile in the central part of the pulse is linear. Away from the central area of the pulse, the frequency chirp deviates from a linear profile. Anomalous dispersion imparts a linear frequency chirp across the entire pulse. It is this non-ideal matching of SPM and dispersive frequency chirp at the wavelength extremities of the pulse that results in the pedestals observed in spectral compression.

To further reduce the level of the pedestals in the spectrally compressed pulses, one possible solution is to implement the self-phase modulation stage in a waveguide with large normal dispersion. This serves to linearize the self-phase modulation frequency chirp profile. Numerical simulations shown in Figure S5 reveal that the spectrally compressed pulse pedestals become smaller when the nonlinear waveguide stage possesses normal dispersion. The spectral compressor (*Device 2*) is modelled using 480 fs sech^2^ pulses at an input pulse peak power of 18 W, with different values of waveguide dispersion (*β*_2_ = -0.2, 0.2, 2, 20 and 40 ps^2^ m^-1^). From Fig. S6a, it may be observed that while the pulse pedestals may be reduced further by 2.5 dB if *β*_2_ is increased from -0.2 ps^2^ m^-1^ to 40 ps^2^ m^-1^.

A similar phenomenon is observed in temporal compression: It is observed from Fig. S6b that the level of the pulse pedestals decrease as the dispersion in the USRN waveguide becomes increasing normal.

**Figure S6**. Numerical analysis of the compressed pulse profile. **a** Numerically calculated spectral compressor output for 480 fs sech^2^ pulses at with *P*_peak(in)_ = 18 W (pulse energy = 8.64 pJ), as a function of the dispersion, *β*_2_ in the USRN waveguide. The level of the pedestals is shown to decrease when the dispersion of the waveguide becomes increasingly normal. **b** Numerically calculated temporal compressor output for 5.8 ps sech^2^ pulses with *P*_peak(in)_ = 13 W (pulse energy = 75.4 pJ), as a function of the dispersion, *β*_2_ in the USRN waveguide. The level of the pedestals is shown to decrease when the dispersion of the waveguide becomes increasing normal.

**References**

1. Tan, D. T. H., Sun P. C. & Fainman, Y. Monolithic nonlinear pulse compressor on a silicon chip. *Nat. Commun.* **1,** 116, (2010).
2. Tsang H. K. et al. Optical dispersion, two-photon absorption and self-phase modulation in silicon waveguides at 1.5 µm wavelength. *Appl. Phys. Lett.* **80,** 416–418 (2002).
3. Tan, D. T. H. Optical pulse compression on a silicon chip: Effect of group velocity dispersion and free carriers. *Appl. Phys. Lett.* **101,** 211112 (2012).
4. Sohn, B.-U., Choi, J. W., Ng, D. K. T. & Tan, D. T. H. Optical nonlinearities in ultra-silicon-rich nitride characterized using z-scan measurements. *Sci. Rep.* **9,** 10364 (2019).
5. Wang T. et al. Supercontinuum generation in bandgap engineered, back-end CMOS compatible silicon rich nitride waveguides. *Laser Photon. Rev.* **9,** 498-506 (2015).
6. Blanco-Redondo A. et al. Observation of soliton compression in silicon photonic crystals. *Nat. Commun.* **5,** 3160 (2014).
